# Childhood maltreatment and NSSI in prisoners: mediation through self-identity and moderation by sensation-seeking

**DOI:** 10.3389/fpsyt.2024.1303033

**Published:** 2024-02-02

**Authors:** Juan Li, Honglei Gu, Tiansheng Xia

**Affiliations:** ^1^ School of Educational Science, Hunan Normal University, Changsha, China; ^2^ Cognition and Human Behavior Key Laboratory of Hunan Province, Hunan Normal University, Changsha, China; ^3^ School of Art and Design, Guangdong University of Technology, Guangzhou, China

**Keywords:** non-suicidal self-injury, childhood maltreatment, sensation-seeking, self-identity, moderated mediation model

## Abstract

**Introduction:**

The prevalence of non-suicidal self-injury (NSSI) among prisoners is widely recognized. However, most research conducted in prisons setting has focused on lifetime engagement in NSSI, with limited knowledge about its correlates and risk factors during imprisonment.

**Methods:**

Drawing on the integrated theoretical model of NSSI, this study aimed to examine the combined effects of childhood maltreatment (an environmental factor) and two intrapersonal factors, namely self-identity and sensation-seeking, on NSSI. 1042 Chinese male offenders participated in the current study, and regression analyses is used to examine the relationship among variables.

**Results:**

The results revealed that self-identity mediated the association between childhood maltreatment and prisoners’ NSSI. Sensation-seeking moderated the relationship between self-identity and NSSI, and this connection was only significant for offenders with high sensation-seeking.

**Discussion:**

We discuss the results of the current research and possible practical implications.

## 1 Introduction

Nonsuicidal self-injury (NSSI) is defined as the intentional and direct damaging or modifying of body tissue without any conscious intent of causing death, and it is considered socially unacceptable (1). This behavior is a pervasive problem in jails and prisons (2), but it has not received enough attention in prevention strategies in many countries. Previous research indicated that incarcerated individuals displayed a higher prevalence of NSSI with estimates suggesting that between 7% and 48% of offenders have committed NSSI (3). In comparison, the prevalence of NSSI among adults in the general population is around 5.5% (4). For example, in Asian areas, the researchers reported a significantly higher prevalence of NSSI among adolescent prisoners, with 73.7% of them engaging in NSSI. By contrast, the control group had a prevalence rate of 17.1% (5). Studies have shown that among the prisoners who commit suicide, about 50% of those have engaged in NSSI while they are incarcerated. This history of NSSI significantly increases the probability of suicide in prison by 6-11 times (6).

The literature has found that early experiences of maltreatment and psychological characteristics are the main reasons for individuals’ NSSI [e.g (7, 8).,]. Nevertheless, its occurrence mechanism is still unclear. Therefore, this study mainly explores the adverse influence of childhood maltreatment on the self-injury behavior among prisoners, as well as the underlying mechanism in order to provide targeted opinions and suggestions for the prevention of, and interventions for, NSSI behavior in prisons.

### 1.1 Childhood maltreatment and NSSI

Childhood maltreatment refers to the physical and emotional neglect or abuse and sexual trauma suffered by an individual during childhood (9). The interpersonal/system model of NSSI supposes that self-injury is symptomatic of family or environmental dysfunction, and the family environment might inadvertently support or reinforce NSSI behavior, which may explain why childhood experiences of maltreatment can predict NSSI behavior (10). The developmental psychopathological framework suggests that individuals develop motivation, attitude, tools, emotions, and interpersonal skills in the process of positive adaptation, and childhood maltreatment will hinder the development of these five abilities. Due to the lack of access to essential adaptive skills and resources during the critical developmental period, individuals may be compelled to seek alternative methods (such as non-suicidal self-injury) to tackle developmental challenges. That is, NSSI is a compensation strategy formed in the process of individual growth (11).

Previous studies documented that childhood maltreatment was related to prisoners’ NSSI [e.g (12, 13).,]. For example, Howard et al. (14) reported that childhood trauma was more prevalent in prisoners with an experience of self-harm than non-self-harm prisoners. Ford et al. (15) found that the prevalence of lifetime self-harm in prison increased with exposure to adverse childhood experiences (ACEs). Specifically, among individuals who have not experienced any ACEs, only 2.7% reported engaging in self-harm while in prison. In contrast, among those who have experienced four or more ACEs, the percentage of individuals who reported lifetime self-harm in prison was significantly higher at 31.0%.

Although previous research supported the relationship between childhood maltreatment and NSSI (13, 15), the underlying mechanism of this association is a subject of debate at present. That is, why individuals choose NSSI instead of other maladaptive coping methods, such as alcohol and drug abuse, when they suffer from childhood maltreatment is unknown. One possible explanation is that individuals choose NSSI as a palliative method due to a lack of self-identity synthesis. Recent studies have indicated that NSSI is closely associated with identity confusion [e.g (16).,], and individuals may commit NSSI in order to gain group identity and offset the adverse impact of self-loss (17). In addition, for prisoners, the level of sensation-seeking may affect the incidence of NSSI, which is an important moderating variable (18). Therefore, this study took self-identity as a mediator and sensation-seeking as a moderator to explore the influence mechanism of childhood maltreatment on NSSI behavior.

### 1.2 Self-identity as a mediator

According to Erikson (19), self-identity can be defined as a personal and subjective sense of remaining unchanged and consistent throughout different periods and situations. It encompasses the understanding of oneself as an individual with a distinct identity, where the past, present, and future aspects of one’s existence are interconnected and intertwined. Self-identity is specifically related to personal experiences and forms one’s view of oneself (17), and thus self-identity has an important impact on subsequent behavior (20).

Erikson’s theory of psychosocial development (19) posits that the family environment (e.g., parenting style, parent-child interaction, childhood maltreatment) significantly influences the formation of self-identity and even develops different styles of self-identity. Numerous empirical evidences have revealed that a lack of self-identity is related to emotional neglect and emotional abuse [e.g (21, 22).,]. Experiences of maltreatment can disrupt individuals’ life plan, and later their life, in unpredictable and sometimes permanent ways (23, 24), and childhood maltreatment has an important influence on the formation of self-identity (25).

Previous studies have found that the lack of self-identity is associated with NSSI (26, 27). The cross-sectional study has demonstrated that a low sense of identity could positively predict NSSI (28), and this view is supported by another longitudinal study (29). In the prison setting, prisoners may engage in NSSI to separate individuals from other people, which sets interpersonal boundaries, and it is beneficial for individuals to assert their identity (30). Individuals who engage in NSSI may use it as a coping mechanism to deal with emotional pain, feelings of emptiness, or a lack of control over their lives. By engaging in self-harm, they create a physical sensation that can distract them from emotional distress or numb their feelings temporarily. This act of self-harm can provide a sense of control and autonomy that might be lacking in other aspects of their lives (30). In addition, interpersonal boundaries caused by NSSI help define and protect their individuality, personal space, and emotional well-being, which contributes to a sense of self-identity (25). It helps individuals differentiate themselves from others and establish their own values, interests, and personal space. By respecting and maintaining these boundaries, individuals can promote healthier relationships and enhance their self-esteem and self-worth.

The benefit and barriers model of NSSI believed that if NSSI behavior can fulfill individual’s positive functions, and then it was likely to be reinforced (31). While they are incarcerated, prisoners are under great pressure from the environment and management, and they are treated in a standardized and homogeneous manner, and they may resort to self-harm to maintain their self-identity and separate themselves from others, which may lead to more self-harm behaviors. However, Hooley and Franklin (31) proposed that a positive view of the self can provide a barrier against NSSI. Gandhi, et al. (32) surveyed 528 high school students, and found that self-identity played a mediating role between behavior inhibition and NSSI, and promoting the integration of self-identity is beneficial to the management of NSSI. These theories and related studies implied that self-identity may be an important mediator to explain the influence of childhood maltreatment on NSSI.

### 1.3 Sensation-seeking as a moderator

Sensation-seeking is a personality trait, which refers to individuals’ pursuit of changeable, new, complex and strong feelings and experiences, and their desire to realize these experiences through taking physiological, social, legal and economic risky behaviors (33). The sensation-seeking model suggests that individuals may conceptualize NSSI as a way of seeking excitement or exhilaration, similar to activities like skydiving or bungee jumping (30). Nixon et al.’ (34) study revealed that 7.1% of the hospitalized adolescents reported engaging in NSSI with the intention of seeking excitement.

Sensation-seeking is related to self-identity. A longitudinal study has found that sensation-seeking increases between the ages of 15 and 24, a period that coincides with the development of individual self-identity (35). Hence, when individuals with high sensation-seeking encounter frustration in their development self-identity, they are likely to adopt more negative behaviors (33). For example, Yu et al. (36) discovered that the experience of cyberbullying victimization, when combined with sensation-seeking tendencies, can influence an individual’s engagement in NSSI. Sensation-seeking was likely to amplify the impact of personal adversity on the risky behaviors (37, 38). Individuals with high sensation-seeking tend to perceive lower potential consequences for engaging in risky behaviors, and they are more willing to engage in NSSI in order to experience the associated benefits, such as creating excitement or finding relief from boredom (39). Additionally, the negative impact of low self-identity on prisoners is significantly amplified by sensation-seeking. When the prisoners fail to establish sense of themselves, those with high sensation-seeking tendencies are more prone to impulsively resort to maladaptive coping strategies, which further increases the likelihood of engaging in NSSI. By contrast, prisoners with low sensation-seeking can objectively judge the possible consequences of risky behavior, and are less likely to regard NSSI as an excitement-regulation strategy. Therefore, sensation-seeking may strengthen the relationship between self-identity and NSSI.

### 1.4 The current study

**Figure 1:**
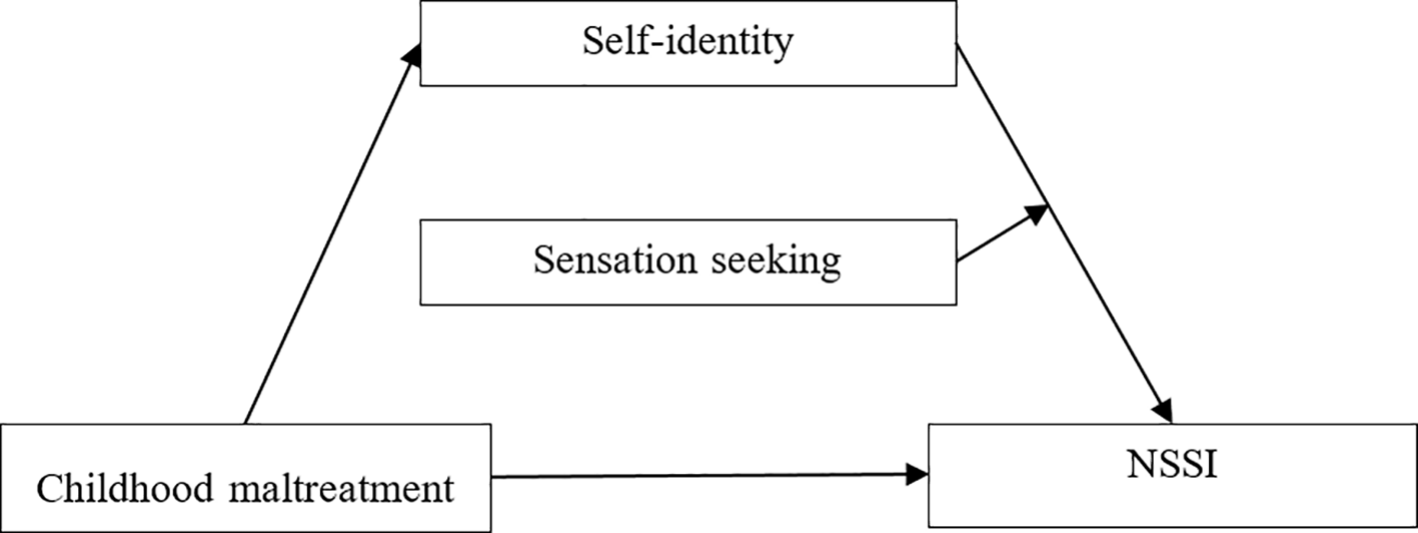
Hypothesized model.

Nock (1) proposed the integrated theoretical model of NSSI, and suggested that early traumatic experiences may have positive effects on individual susceptibility factors, which work together with an individual’s emotional genetics to cause NSSI. Hence, based on Erikson’s personality development theory and the integrated theoretical model of NSSI, this study intends to test a moderated mediation model and investigates the joint influence of one environmental factor of childhood trauma and two intrapersonal factors of self-identity and sensation-seeking on prisoners’ NSSI. Therefore, two hypotheses were proposed (see **Figure 1**):

H1. Self-identity will mediate the association between childhood maltreatment and NSSI within the prison population;

H2: Sensation-seeking will moderate (i.e., strengthen) the relationship between self-identity and NSSI within the prison population.

## 2 Materials and methods

### 2.1 Participants

1042 male prisoners (*M*_age_ = 38.45, *SD* = 10.67) participated in this study. These participants were incarcerated in two prisons in China. In this sample, 440 (42.2%) had been convicted of violent crimes. Most of them (*n* = 651, 62.5%) reported being from rural areas, which typically have lower income and educational levels compared to urban areas in China. Before being incarcerated, almost half of the participants (49.0%) were married or in a cohabiting relationship. Thirty percent of the participants were single, and the rest (21.0%) were either divorced or widowed. The average duration of time spent in prison was 63.66 ± 40.60 months, ranging from 12 to 228 months.

### 2.2 Measures

In the correctional environment, we evaluated non-suicidal self-injury (NSSI) using the following question: “Within the previous one year, have you purposely harmed yourself without the intention of suicide by burning yourself, stabbing your own skin, scratching your skin, hitting your head, cutting yourself, or biting yourself?” This question was adapted from the Inventory of Statements About Self-Injury [ISAS (40);] and the interviewer rated each item on a 4-point Likert scale: 1 meant never, 2 meant once or twice, 3 meant three to five times, and to 4 meant six times or more. The lowest score on the questionnaire was 4, and the highest was 24, with higher scores indicating higher frequency of NSSI. In this study, the measure had a Cronbach’ α of 0.87.

Childhood maltreatment was assessed by the Childhood Trauma Questionnaire -short developed by Bernstein et al. (41). This questionnaire is composed of five components: emotional abuse, physical abuse, sexual abuse, emotional neglect, and physical neglect. Each dimension comprises five items, such as “Being beaten so severely that it was noticed by a teacher/neighbor/doctor.” Participants rated each item on a 5-point Likert scale, ranging from 1 (never true) to 5 (very often true). We calculated the average score of the participants on each item, and their average scores were used to determine the level of childhood maltreatment. This questionnaire was translated and revised by the Chinese researchers and was verified to be a reliable and valid measure [e.g (42).,]. In current study, the Cronbach’s α for the questionnaire was 0.88.

Sensation-seeking was measured by a subset of six items from the sensation-seeking scale [SSS (43);], as employed by Steinberg et al. (44). These items specifically capture the inclination towards seeking thrills or novelty, rather than impulsivity. An example item is “I sometimes enjoy engaging in activities that are a little frightening.” Participants rated each item on a 5-point Likert scale, and 1 meant strongly disagree, 5 meant strongly agree. The final score was obtained by calculating the average score for all items, with higher scores indicating a greater inclination towards sensation-seeking. This measure has previously been validated in Chinese samples (45). In current study, the Cronbach’s α coefficient for the measure was 0.76.

The Identity and Identity Diffusion Scale (IIDS), which was developed by Ochse and Plug (46), was used to assess self-identity. This scale comprised 19 items, with a sample item being “I feel certain about what I should do with my life.” Participants were asked to indicate their level of agreement with each statement on a 5-point Likert scale, ranging from 1 (strongly disagree) to 5 (strongly agree). For items with negative statements, the responses were reverse-coded. We calculated the average score of the participants on each item, and used them to determine the level of sense of identity. Several studies have documented the reliability and validity of this scale [e.g (47).,]. In the present study, the Cronbach’s α coefficient for the scale was 0.69.

### 2.3 Procedures

The current research was approved by the Departmental Ethics Committee and the Institutional Review Board of the Guangdong University of Technology. Based on geographical proximity, two prisons were selected. 1131 male prisoners were involved in this study. After excluding 89 participants who indiscriminately selected the same response to all items, the final sample size was reduced to 1042. Prior to their involvement, all participants completed written informed consent. In the current study, prisoners did not receive any incentives for their participation.

This study was carried out in two sessions. First, prisoners filled out questionnaires related to demographics, childhood trauma, psychopathy, and cognitive reappraisal. These questionnaires were completed in groups of around 12-16 individuals in a calm room. A trained researcher was present to supervise the session and address any queries participants had regarding the questionnaires or the study. Subsequently, participants underwent an interview-based assessment of NSSI. This assessment was conducted by master’s-level students in clinical psychology in a private assessment room.

### 2.4 Data analysis

The first step involved calculating descriptive statistics and Pearson correlations among the variables in the study. This allowed us to understand the relationships between the variables. Next, we utilized Model 4 of Hayes’s PROCESS macro (48) to investigate whether self-identity mediated the link between childhood maltreatment and NSSI. In the third step, we used Model 14 of the PROCESS macro to conduct a moderated mediation analysis. This analysis aimed to examine whether the indirect path between childhood maltreatment and NSSI was influenced by sensation-seeking.

To assess the significance of the effects in Model analysis, we used bootstrap confidence intervals (CIs) based on 5000 random samples. If the CIs did not include zero, the effects were considered significant. It’s important to note that all study variables were standardized in both Model 4 and Model 14 before conducting the data analyses. To account for the influence of age and the classification of crimes on prisoners’ NSSI, we included these demographic variables as control variables. Age was treated as a continuous variable, meaning that we considered the precise numerical value of each participant’s age. On the other hand, the classification of crimes was considered as a dichotomous variable. We assigned a code of 0 to indicate non-violent crimes and a code of 1 to indicate violent crimes. By controlling for these variables, we aimed to isolate the specific effects of other factors on prisoners’ NSSI, while accounting for the potential influence of age and the classification of crimes. Previous studies by Knight et al. (49) and Smith and Kaminski (50) have highlighted the significance of these factors in understanding prisoners’ NSSI.

## 3 Results

### 3.1 Preliminary analyses

As presented in **Table 1**, childhood maltreatment and sensation-seeking were found to have negative correlations with self-identity, and positive correlations with NSSI. Furthermore, self-identity was found to have a negative correlation with NSSI. In terms of demographic variables, age was found to have a negative correlation with NSSI and self-identity. Additionally, the classification of crimes was found to have a positive correlation with NSSI and a negative correlation with self-identity. This indicates that individuals involved in violent crimes are more likely to engage in NSSI behaviors and may have a weaker sense of self-identity compared to those involved in non-violent crimes. These correlations provide valuable insights into the relationships between the variables and contribute to our understanding of the factors influencing NSSI among prisoners.

**Table 1:** Means, standard deviations and correlations of the study variables.

	1	2	3	4	5	6
1. Age	—					
2. Classification of crimes	–0.27^***^	—				
3. Childhood maltreatment	–0.07^*^	0.15^***^	—			
4. Self-identity	–0.12^***^	–0.09^**^	–0.24^***^	—		
5. Sensation-seeking	–0.16^***^	0.08^*^	0.14^***^	–0.19^***^	—	
6. NSSI	–0.10^**^	0.08^*^	0.34^***^	–0.22^***^	0.15^***^	—
*M*	38.45	0.46	1.52	3.18	2.75	1.09
*SD*	10.67	0.50	0.48	0.43	0.81	0.33

n = 1042. Classification of crimes was coded as 0 = non-violent crime and 1 = violent crime. ^*^ *p* < 0.05, ^**^ *p* < 0.01, ^***^ *p* < 0.001.

### 3.2 Testing for the mediation effect of self-identity

We utilized Model 4 of the PROCESS macro to test the mediation effect of self-identity in the relationship between childhood maltreatment and NSSI. After controlling for age and the classification of crimes, the results from **Table 2** revealed several significant associations. First, childhood maltreatment was found to be negatively associated with prisoners’ self-identity (*b* = –0.23, *t* = –7.34, *p* < 0.001). Furthermore, self-identity was found to be negatively related to prisoners’ NSSI (*b* = –0.14, *t* = –4.34, *p* < 0.001). Importantly, even after accounting for the mediating effect of self-identity, a significant positive direct association between childhood maltreatment and NSSI remained (*b* = 0.32, *t* = 10.04, *p* < 0.001). This suggests that childhood maltreatment has a direct impact on NSSI, independent of its influence on self-identity. To further examine the mediating role of self-identity, bootstrapping analyses were conducted. The results indicated that self-identity significantly mediated the relationship between childhood maltreatment and prisoners’ NSSI, with an indirect effect of 0.04 (*SE* = 0.01, 95%CI = [0.02, 0.05]). This means that approximately 11.1% of the total effect can be accounted for by the mediating effect of self-identity. Based on these findings, Hypothesis 1 was supported, suggesting that self-identity mediates the effect of childhood maltreatment on NSSI among prisoners.

**Table 2:** Testing the mediation effect of self-identity.

Predictors	Model 1 (NSSI)		Model 2 (Self-identity)		Model 3 (NSSI)	
	*b*	*t*	*b*	*t*	*b*	*t*
Age	–0.01	–2.55^*^	0.01	3.43^***^	–0.01	–2.07^*^
Classification of crimes	0.01	0.18	–0.06	–0.92	0.00	0.05
Childhood maltreatment	0.36	11.25^***^	–0.23	–7.34^***^	0.32	10.04^***^
Self-identity					–0.14	–4.34^***^
*R* ^2^	0.13		0.08		0.15	
*F*	47.47^***^		25.62^***^		40.98^***^	

*p < 0.05, ***p < 0.001.

### 3.3 Testing for moderated mediation

**Figure 2:**
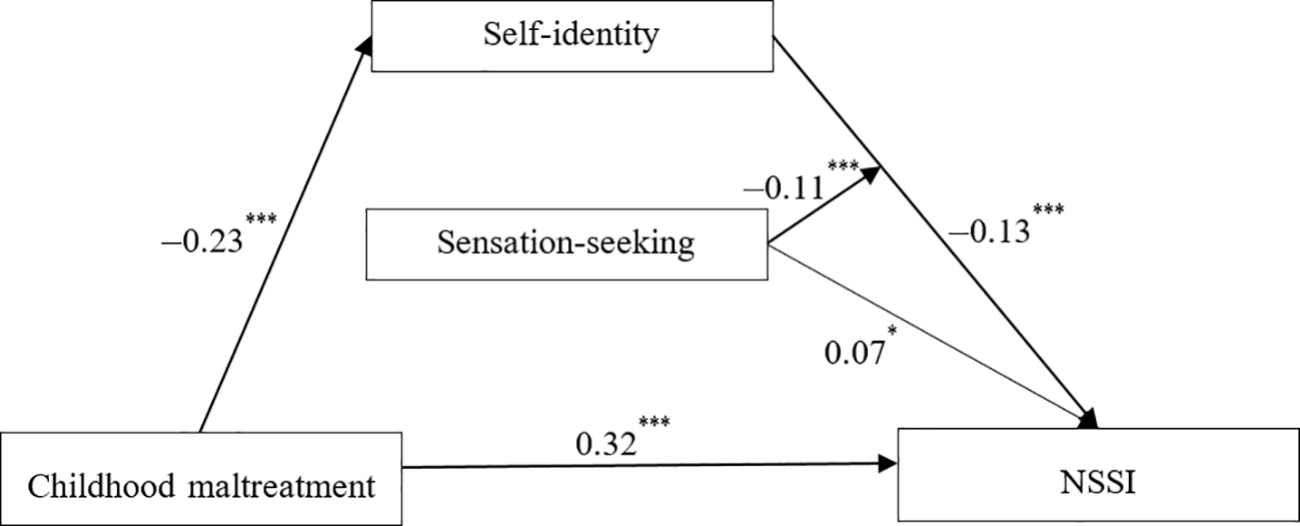
Unstandardized path coefficients for the moderated mediation model for (NSSI). For simplicity, the effects of demographics on self-identity and NSSI were not displayed. *p < 0.05, ^***^ *p* < 0.001.

We utilized Model 14 of Hayes’s PROCESS macro to test the moderation effect of sensation-seeking on the indirect relations between childhood maltreatment and NSSI. The results, as depicted in **Figure 2**, revealed a significant predictive effect of the interaction term between self-identity and sensation-seeking on NSSI. Specifically, the product or interaction term of self-identity and sensation-seeking was significantly correlated with NSSI (*b* = –0.11, *t* = –3.96, *p* < 0.001). This indicates that the association between self-identity and NSSI is influenced by individuals’ levels of sensation-seeking.

**Figure 3:**
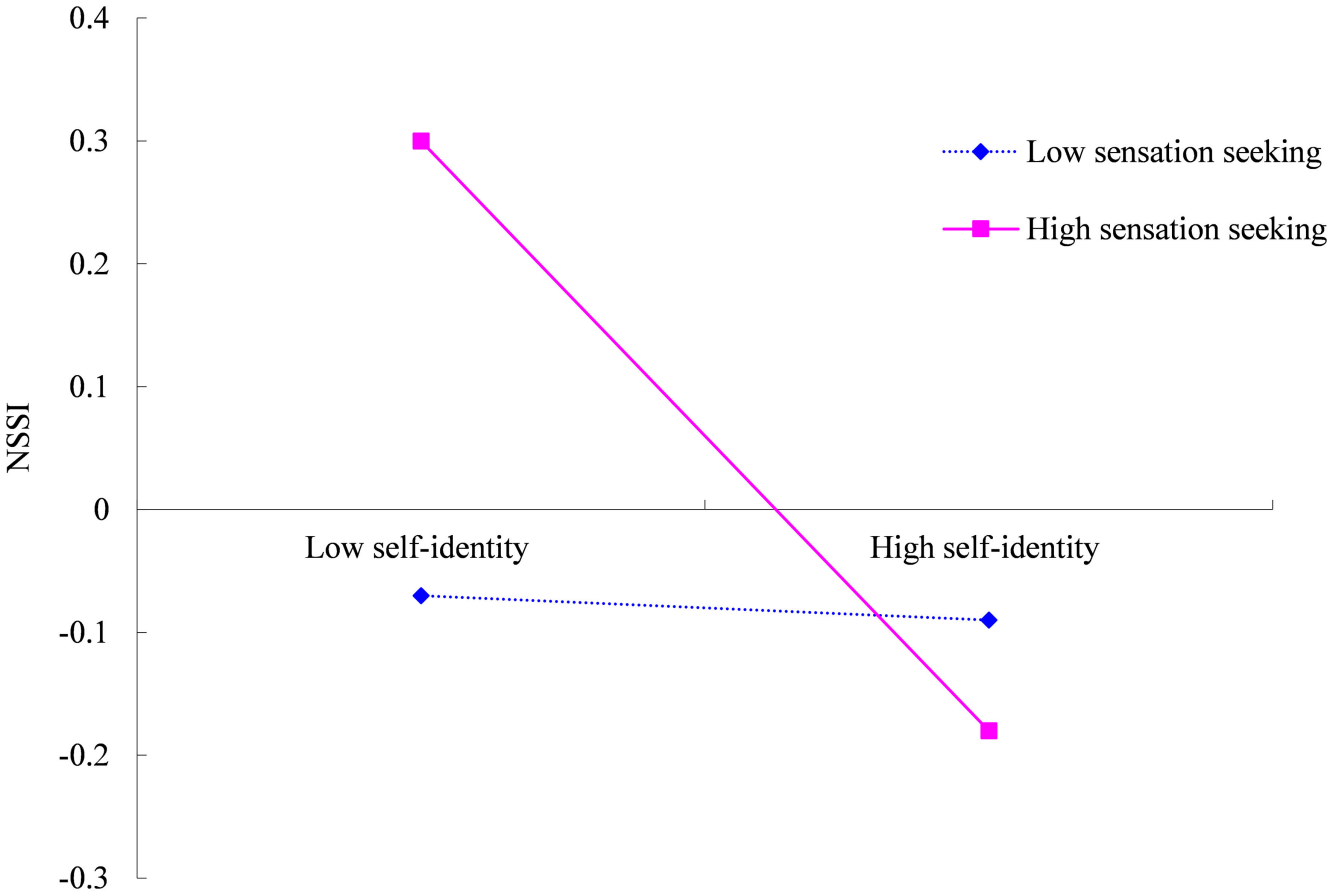
Interactive effect of sensation-seeking and self-identity on NSSI.

To further understand this interaction effect, simple slope analyses were conducted (see **Figure 3**). For individuals with high levels of sensation-seeking (*M+SD*), self-identity was strongly negatively related to NSSI (*b*_simple_ = –0.24, *t* = –5.62, *p* < 0.001). However, for individuals with low levels of sensation-seeking (*M-SD*), the negative relationship between self-identity and NSSI was weaker and non-significant (*b*_simple_ = –0.02, *t* = –0.39, *p* > 0.05). These findings suggest that sensation-seeking strengthens the association between self-identity and NSSI.

The results of the conditional indirect effects analysis revealed that the indirect effect of childhood maltreatment on NSSI through self-identity was moderated by sensation-seeking. Specifically, for prisoners with high levels of sensation-seeking (*M+SD*), there was a significant indirect relationship between childhood maltreatment and NSSI (indirect effect = 0.06, *SE* = 0.02, 95% CI = [0.02, 0.09]). However, for prisoners with low levels of sensation-seeking (*M-SD*), the indirect relationship between childhood maltreatment and NSSI was no longer significant (indirect effect = 0.00, *SE* = 0.01, 95% CI = [–0.01, 0.02]). These findings suggest that individuals with high sensation-seeking may be more susceptible to the indirect effects of childhood maltreatment on NSSI through self-identity.

## 4 Discussion

While there is substantial empirical evidence supporting the relationship between childhood maltreatment and NSSI, the specific mechanisms that mediates and moderates this relationship are still not well understood. To address this gap, we constructed a moderated mediation model and found that childhood maltreatment was positively related to NSSI among prisoners, and self-identity mediated this relationship. Furthermore, sensation-seeking amplified the effect of self-identity on NSSI.

### 4.1 The mediation effect of self-identity

Our study revealed a significant positive prediction effect of childhood maltreatment on NSSI, suggesting that prisoners who have experienced childhood maltreatment are more prone to engaging in NSSI. This finding aligns with previous research findings on the subject (2, 13). This result is also consistent with the interpretation of the developmental psychopathology model, which suggests that childhood maltreatment prevents individuals from acquiring the necessary adaptability in critical periods, so NSSI is used as a maladaptive response to cope with developing problems (11).

More importantly, we found that self-identity was a mediating factor between childhood maltreatment and prisoners’ NSSI; that is, the frequent experience of childhood maltreatment will have a negative influence on the formation of self-identity and a lack of self-identity further promotes the use of NSSI to cope with their emotional problems. In other words, the formation of self-identity is one of the important mechanisms by which childhood maltreatment influences the NSSI behavior of prisoners. This finding provides a possible explanation for the association between childhood maltreatment and NSSI from the perspective of the Erikson theory of psychosocial development. NSSI is an inadaptable coping style of prisoners in the face of childhood maltreatment and environmental stress, which is partly derived from their lack of identity (51). Because of their low psychological maturity, the development of self-identity will be seriously affected when the individual is in a high-conflict family atmosphere. In the face of the experience of childhood maltreatment, individuals who did not form a strong self-identity tend to be more uncertain about their own values and outlook on life, which causes them to make incorrect attributions regarding the experience of abuse, or adopt unhelpful impulses, such as avoiding the adverse situation temporarily through NSSI. Negative experiences can be temporarily eliminated or alleviated, and individuals can temporarily feel relaxed, released and calm, thus achieving emotional regulation and reducing psychological pain (52).

Recent studies found that childhood maltreatment affects individuals’ maladaptive behaviors through self-identify, including not only self-injures, but also adult couple adjustment (22). Self-identity deficits caused by child emotional abuse can have a negative impact on female couple adjustment (22). In addition to childhood maltreatment, cultural environment may influence self-identity and contribute to maladaptive coping mechanisms, including self-injury. For example, Gandhi et al. (53) reported that compared to the Indian sample, Belgian participants were more likely to resort to self-injury for intra-personal reasons, and the associations between NSSI and identity were also stronger.

### 4.2 The moderation effect of sensation-seeking

Our finding showed that sensation-seeking moderated the relationship between self-identity and NSSI. Specifically, among prisoners with high sensation-seeking, the influence of self-identity on NSSI was significantly stronger. However, for prisoners with low sensation-seeking, this association between self-identity and NSSI was not significant. These findings can be explained by the sensation-seeking model of NSSI. Based on this view, high sensation-seeking exacerbated the risk effect of a weak self-identity. Specifically, the prisoners with high sensation-seeking were more prone to regard NSSI as a means of excitement or novelty when they failed to establish sense of themselves because of their early negative experiences. Several studies have found that sensation-seeking is significantly positively related to NSSI (18, 37), and individuals with high sensation-seeking tend to have higher participation rates in risky behaviors and achieve self-reward through risky behaviors. The growth period of sensation-seeking is highly coincident with the development of identity (35). When the prisoners failed to establish a sense of self, those with high sensation-seeking were more inclined to take risks and tolerate physical pain and other negative consequences in exchange for the potential rewards. This willingness to accept risks and engage in potentially harmful behaviors lowered the barriers to engaging in NSSI and increased the overall risk of participating in NSSI (39). However, individuals with a lower level of sensation-seeking had a weaker desire for risky behaviors, thus weakening the influence of lack of self-identity on individuals’ risky behaviors. Other studies have also found that sensation-seeking moderated (i.e., strengthened) the indirect effects of childhood maltreatment on other individual problem behaviors, such as problematic mobile phone use (54).

### 4.3 Limitations and implications

When interpreting our findings, several limitations should be noted. First, the use of cross-sectional data restricts our ability to establish causal inferences. We should further employ longitudinal designs to examine the model proposed in this study. Second, although the questionnaires used in this study have demonstrated good reliability and validity in Chinese samples, the retrospective collection of childhood maltreatment data may be susceptible to recall biases, which could impact the validity of our findings. Lastly, the sample in the current study consisted solely of male prisoners from several Chinese prisons. One recent study which adopted 1040 Chinese younger prisoners as the sample found that the effect of childhood maltreatment on NSSI was stronger in females than in males (55). Other researchers also found the similar results (56). Thus the generalizability of our findings to female prisoners or other culture contexts should be further investigated.

The current study found the joint effect of childhood maltreatment, self-identity and sensation-seeking on the NSSI behavior of prisoners, which provides important information regarding the prevention and intervention of prisoners’ NSSI behavior. First, optimizing the living environment and prison-management mode of prisoners can reduce their memories of traumatic childhood experiences, reduce their stimulation of sensory-seeking risk factors, increase their attention to the development of self-identity, and thus prevent the occurrence of NSSI. Second, intervention and guidance regarding the formation of self-identity is also important. Individuals with an integrated self-identity can form positive personality traits, and can alleviate the negative effect of childhood trauma, thus reducing the occurrence of NSSI. Psychotherapy, such as Cognitive-Behavioral Therapy (CBT), can help individuals develop a stronger sense of self and more adaptive coping skills (57). These therapies can provide individuals with tools to examine underlying beliefs about themselves and challenge negative self-perceptions. Third, it is necessary to reduce the individual’s sensation-seeking tendencies. Our findings indicate that low sensation-seeking tendencies may motivate individuals to be less likely to engage in NSSI. This seems to offer the possibility that prison administrators can guide prisoners to reduce sensation seeking and avoid using sensation seeking as an arousal or trigger, especially for individuals within disadvantaged or with low self-identity. Some practices such as mindfulness and meditation can promote greater self-awareness and reduce impulsivity, which can help reduce the tendency to engage in sensation-seeking behaviors (58). Finally, the moderated mediating model constructed in the present study showed that the environment and individuals acted together regarding the NSSI behavior of prisoners. Therefore, in future interventions, it is better to conduct an integrated and systematic intervention instead of focusing on a single aspect, to achieve the best intervention effect and reduce the incidence of NSSI to the greatest extent.

## Data availability statement

The raw data supporting the conclusions of this article will be made available by the authors, without undue reservation.

## Ethics statement

This work has been approved by the Departmental Ethics Committee and the Institutional Review Board of Guangdong University of Technology. The studies were conducted in accordance with the local legislation and institutional requirements. The participants provided their written informed consent to participate in this study.

## Author contributions

JL: Conceptualization, Investigation, Writing – original draft. HG: Conceptualization, Formal Analysis, Writing – original draft. TX: Funding acquisition, Writing – original draft.
